# Application of telepresence systems in teaching – transfer of an interprofessional teaching module on digital aided communication into the block training “internal medicine” during the Covid-19 pandemic

**DOI:** 10.3205/zma001377

**Published:** 2020-12-03

**Authors:** Elisa Haucke, Jens Walldorf, Christiane Ludwig, Christian Buhtz, Dietrich Stoevesandt, Katharina Clever

**Affiliations:** 1 Martin-Luther-Universität Halle-Wittenberg, Medizinische Fakultät, Dorothea Erxleben Lernzentrum, Halle (Saale), Germany; 2 Universitätsklinikum Halle, Universitätsklinik und Poliklinik für Innere Medizin, Halle (Saale), Germany

**Keywords:** telemedicine, educational technology, education, medical education, nursing

## Abstract

**Objective:** The contact restrictions caused by the Covid-19 pandemic fundamentally limit patient-centered teaching. To realize a patient-oriented education in the block training “Internal Medicine” at the University Hospital Halle (Saale) despite the challenges, the already established teaching module “Interprofessional Teleconsultation” was adapted. The short article outlines the interprofessional teaching module including first evaluation results and describes the adapted block training.

**Method:** In the “Internal Medicine” block training, students in a lecture hall navigated a telepresence system, which was accompanied by a physician across the ward and conducted an anamnesis via video and audio transmission without actual patient contact.

**Results: **Students, physicians, and patients were open-minded about this form of communication during the Covid-19 pandemic and quickly got accustomed to the use of the telepresence system. To be able to react to technical challenges (e.g. unstable connection between the communication partners), a careful preparation of the lecturers is necessary.

**Conclusion: **In using a telepresence system, patient-oriented teaching of students in the block training “Internal Medicine” can be ensured with low-threshold technical effort during the Covid-19 pandemic. The telepresence system allows for the involvement of patients into teaching while adhering to the necessary hygiene measures. Despite technical challenges, the teaching format based on telepresence is suitable as an alternative to face-to-face teaching if actual patient contact is not possible.

## Zusammenfassung

**Zielsetzung: **Die Kontakteinschränkungen durch die Covid-19-Pandemie beeinträchtigen die patientennahe Lehre grundlegend. Um trotz der besonderen Herausforderungen eine patientennahe Ausbildung im Blockpraktikum „Innere Medizin“ am Universitätsklinikum Halle (Saale) zu realisieren, wurde das bereits etablierte Lehrmodul „Interprofessionelles Telekonsil“ adaptiert. Der Kurzbeitrag skizziert das interprofessionelle Lehrmodul mit ersten Evaluationsergebnissen und beschreibt das adaptierte Blockpraktikum.

**Methodik:** Im Blockpraktikum „Innere Medizin“ navigieren die Studierenden von einem Hörsaal aus ein von einem*r Ärzt*in begleitetes Telepräsenzsystem über die Station und führen so via Video- und Audio-Übertragung ein Anamnesegespräch ohne unmittelbaren Patientenkontakt.

**Ergebnisse: **Sowohl die Studierenden und Ärzt*innen als auch die Patient*innen standen dieser Form der Kommunikation während der Covid-19-Pandemie aufgeschlossen gegenüber und gewöhnten sich schnell an den Umgang mit dem Telepräsenzsystem. Um gegebenenfalls auf technische Herausforderungen (z.B. instabile Verbindung zwischen den Kommunikationspartner*innen) reagieren zu können, ist eine sorgfältige Vorbereitung der Dozent*innen notwendig.

**Schlussfolgerung: **Durch den Einsatz des Telepräsenzsystems kann die patientennahe Ausbildung der Studierenden im Blockpraktikum „Innere Medizin“ während der Covid-19-Pandemie mit niederschwelligem technischen Aufwand gewährleistet werden. Das Telepräsenzsystem ermöglicht dabei die Einbindung von Patient*innen in die studentische Lehre bei gleichzeitiger Einhaltung der erforderlichen Hygienemaßnahmen. Trotz technischer Herausforderungen ist das Lehrformat mit Telepräsenz als Alternative zur Präsenzlehre geeignet, wenn ein unmittelbarer Patientenkontakt nicht möglich ist.

## Introduction

Telemedicine provides an opportunity to make better use of medical knowledge not only spatially but also cross-sectoral and to ensure comprehensive medical care [1]. During the Covid-19 pandemic, telemedical care has become increasingly important [2]. This article shows how telecommunication was used in the block training “Internal Medicine” during the Covid-19 pandemic (summer semester 2020) to ensure patient-oriented medical education at the University Hospital Halle (Saale; UKH) despite strict contact restrictions.

For this purpose, the already established teaching module “Interprofessional Teleconsultation” [3] was adapted. In the following, the teaching module “Interprofessional Teleconsultation”, including first evaluation results as well as the adapted block internship “Internal Medicine” are presented. The adaptation of a telepresence-based teaching module shows how practical teaching during the Covid-19 pandemic can be realized based on already existing teaching formats in a resource-saving manner.

## Teaching module “interprofessional teleconsultation”

**Figure 1:**
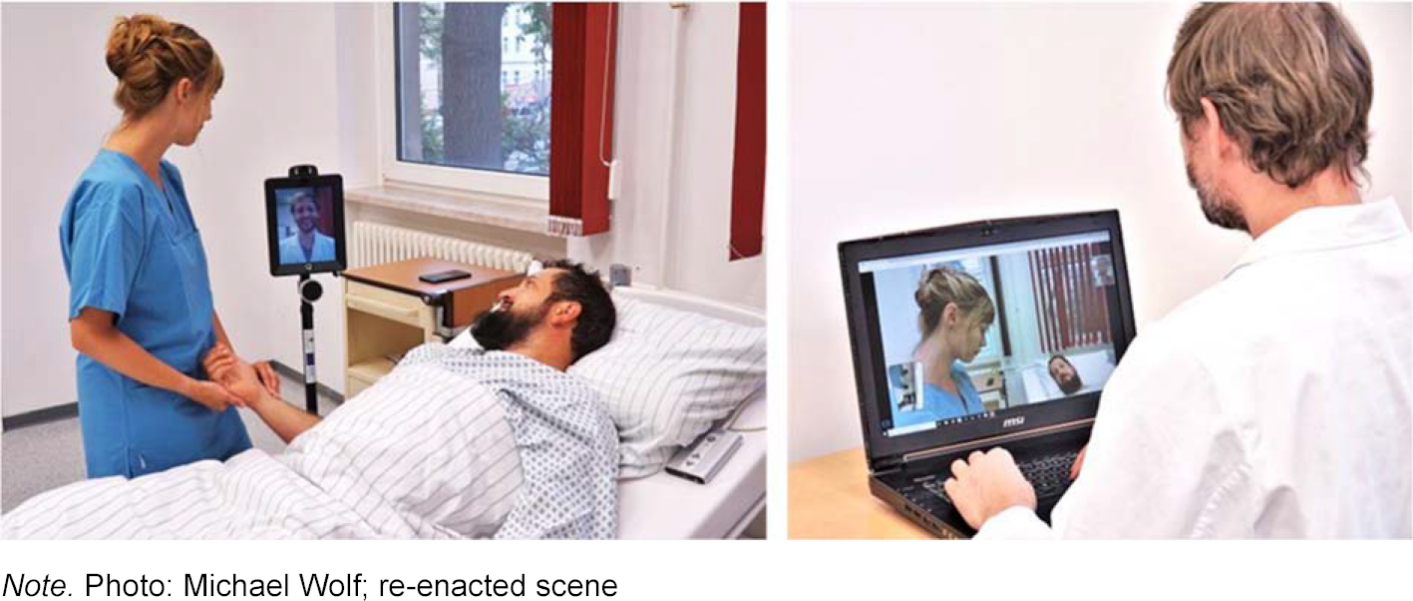
Visit with telepresence system in the teaching module “Interprofessional Teleconsultation”

The teaching module “Interprofessional Teleconsultation” is embedded in an interprofessional teaching series (Project GReTL2.0 – Health Professions in Reflective and Transformative Learning) [4]. Medical and nursing students use a remote-controlled telepresence system (Double2, Double Robotics, USA) in patient conversations and reflect on its use. The mobile telepresence system offers the opportunity to communicate from a distance via video conferencing and to move around in a different environment. After a lecture on the subject of “Digitalization and Assistive Technology”, a simulated visit with an acting patient in an inpatient care facility is prepared in interprofessional groups. The nurse is on site with an immobile resident, while the physician is connected via a telepresence system. The physician navigates the telepresence system via a laptop from a simulated family practice (see figure 1 (Fig. 1)). In a structured feedback session, students reflect on the experienced visit and discuss the possibilities and limits of telepresence systems. 

The piloting of the teaching module was evaluated by 29 students (16 nursing, 13 medicine). The evaluation results indicate a high acceptance of the module procedure and the didactical structure (see table 1 (Tab. 1)). The teaching module thus offers the opportunity of interprofessional sensitization for the use of digital technologies in the professional context.

## Telepresence in the block training “internal medicine”

Contact restrictions due to the Covid-19 pandemic considerably limit patient-based teaching. However, “presence” plays an important role especially in the contact with patients. The teaching module “Interprofessional Teleconsultation” was therefore adapted for teaching in the block training “Internal Medicine”. In comparison to the simulated patient conversation in the teaching module “Interprofessional Teleconsultation”, the data protection officer and the head of information and communication technology at the UKH were involved in the clinical application in order to ensure a secure and stable data transfer.

In each three-hour block training, 20 medical students participated in a lecture hall in compliance with the current hygiene regulations (distance, face masks, hand disinfection). The students accompanied a physician across the ward via video transmission, using a telepresence system and alternately conducted an anamnesis with different patients (approx. 3-4), using the digital medical record. One student was at the laptop and conducted the conversation with the patient, while the other students saw the patient on a screen. To support the conversation, the students also used individual visual, macroscopic aspects for the differential diagnosis. The anamnesis was accompanied and moderated, if necessary, by a second physician in the lecture hall. Patients were informed in advance about the setting. Oral consent was obtained and documented in the patient file.

Overall, both students and physicians appreciated that patient contact was facilitated in the current pandemic situation and quickly got used to the use of the telepresence system. In our experience, patients were also open-minded towards this form of communication. The challenge of using a telepresence system in the “Internal Medicine” block training lies primarily in ensuring a stable connection between the communication partners. Limitations can lead to a loss of quality in the transmission of images and sound. This in turn causes delays in the process and leads to misunderstandings in communication. To be able to meet possible technical challenges ad hoc, careful preparation of the lecturers and the involved department/ward is necessary. 

Despite strict contact restrictions during the Covid-19 pandemic, the use of a telepresence system enables students to actively participate in actual patient conversations while maintaining the necessary hygiene measures to protect the patients. In the future, the telepresence system may also be used in other situations, e.g., to enable students to participate in the rounds of infectious patients. In addition to internal medicine, telepresence-based block trainings are already being carried out at other wards of the UKH (e.g., paediatrics) or as outpatient trainings (e.g., orthopaedics, reproductive medicine, urology). Despite technical challenges, a teaching format that includes a telepresence system is suitable as an alternative to face-to-face teaching if actual patient contact is not possible.

## Competing interests

The authors declare that they have no competing interests. 

## Figures and Tables

**Table 1:**
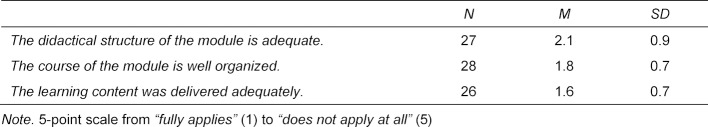
Evaluation of the organisation of the teaching module “Interprofessional Teleconsultation”
